# Single-Trial Event-Related Potential Correlates of Belief Updating

**DOI:** 10.1523/ENEURO.0076-15.2015

**Published:** 2015-10-15

**Authors:** Daniel Bennett, Carsten Murawski, Stefan Bode

**Affiliations:** 1 Melbourne School of Psychological Sciences, The University of Melbourne, Parkville, Victoria 3010, Australia; 2 Department of Finance, The University of Melbourne, Parkville, Victoria 3010, Australia

**Keywords:** belief updating, computational modeling, learning, P3, single-trial, SPN

## Abstract

Belief updating—the process by which an agent alters an internal model of its environment—is a core function of the CNS. Recent theory has proposed broad principles by which belief updating might operate, but more precise details of its implementation in the human brain remain unclear. In order to address this question, we studied how two components of the human event-related potential encoded different aspects of belief updating. Participants completed a novel perceptual learning task while electroencephalography was recorded. Participants learned the mapping between the contrast of a dynamic visual stimulus and a monetary reward and updated their beliefs about a target contrast on each trial. A Bayesian computational model was formulated to estimate belief states at each trial and was used to quantify the following two variables: belief update size and belief uncertainty. Robust single-trial regression was used to assess how these model-derived variables were related to the amplitudes of the P3 and the stimulus-preceding negativity (SPN), respectively. Results showed a positive relationship between belief update size and P3 amplitude at one fronto-central electrode, and a negative relationship between SPN amplitude and belief uncertainty at a left central and a right parietal electrode. These results provide evidence that belief update size and belief uncertainty have distinct neural signatures that can be tracked in single trials in specific ERP components. This, in turn, provides evidence that the cognitive mechanisms underlying belief updating in humans can be described well within a Bayesian framework.

## Significance Statement

Recent theories propose that a central function of the brain is belief updating, the process by which internal models of the environment are revised. However, despite strong implications for cognition, the neural correlates of belief updating remain poorly understood. This study combined computational modeling with analysis of the event-related potential (ERP) to investigate neural signals, which systematically reflect belief updating in each trial. We found that two ERP components, P3 and stimulus-preceding negativity, respectively encoded belief update size and belief uncertainty. Our results shed light on the implementation of belief updating in the brain, and further demonstrate that computational modeling of cognition in ERP research can account for variability in neural signals, which has often been dismissed as noise.

## Introduction

In an uncertain and dynamically changing world, survival depends upon having accurate beliefs about the environment. The more accurately an agent’s beliefs predict environmental contingencies such as threats from predators or the availability of food, the more effectively the agent can plan its actions (Gläscher et al., 2010; Wunderlich et al., 2012). In particular, where environmental contingencies are unknown or nonstationary, an agent should constantly update beliefs in order to produce adaptive behavior (Behrens et al., 2007). Belief updating has generally been studied within a Bayesian framework (Nassar et al., 2010; Stern et al., 2010), wherein beliefs are described by probability distributions over possible states of the world. Bayesian belief updating is captured by the transformation of prior beliefs into posterior beliefs after new information is observed (Knill and Pouget, 2004; Courville et al., 2006).

Recent theories propose that belief updating may be a general principle underlying neural functioning, not merely an adaptive feature of cognition (Fiorillo, 2008, 2012; Friston, 2010). This hypothesis has strong implications for the understanding of human cognition (Bubic et al., 2010; Schwartenbeck et al., 2013). However, while general computational principles of belief updating are well understood, details of the mechanisms by which belief updating is performed in the human brain remain unclear. In addition, some recent research has suggested that the ability of decision makers to update beliefs in a Bayes-optimal fashion may depend on the complexity of the decision situation and on the availability of heuristic alternatives to Bayesian updating (Achtziger et al., 2014, 2015). The present study addressed these questions by comparing Bayesian and heuristic accounts of belief updating, and by assessing how Bayesian belief updating was associated with two event-related potential (ERP) components typically linked with prediction and learning: the P3 and the stimulus-preceding negativity (SPN).

These components are implicated in belief updating by their association with learning and prediction. The P3 is a positive ERP component, the amplitude of which indexes the information content or surprise of an eliciting stimulus (Sutton et al., 1967; Mars et al., 2008). Under the context-updating hypothesis, P3 amplitude is thought to reflect the updating of internal schemata representing stimulus context (Donchin and Coles, 1988). These functions are broadly compatible with belief updating in the Bayesian sense of the term (Kopp, 2008). Furthermore, Mars et al. (2008) hypothesized that a fronto-central subcomponent of the P3 (the P3a; Polich, 2007) encodes belief update size. The present study explicitly tested this hypothesis.

The SPN is a negative-going slow wave elicited by stimulus anticipation (Brunia, 1988). SPN amplitude increases prior to stimuli delivering response reinforcement, both for reward (Masaki et al., 2010) and for instructive feedback (Morís et al., 2013), and covaries with the predictability and expected information of feedback (Kotani et al., 2003; Catena et al., 2012). The present study investigated whether SPN amplitude was related to belief uncertainty prior to updating.

We recorded the electroencephalogram (EEG) from participants performing a perceptual learning task with monetary feedback and used a Bayesian framework to estimate participants’ beliefs at each trial. Model-derived variables related to belief updating were then used to regress single-trial variations in ERP components (Bénar et al., 2007; Mars et al., 2008; van Maanen et al., 2011; Ostwald et al., 2012; Lieder et al., 2013; Kolossa et al., 2015).

## Materials and Methods

### Participants

Participants were 18 right-handed individuals with normal or corrected-to-normal visual acuity. Human subjects were recruited from among the staff and students of The University of Melbourne. The exclusion criterion was a medical history of any neurological disorder, including migraine and epilepsy. Informed consent was acquired from all participants in accordance with the Declaration of Helsinki, and approval was obtained from The University of Melbourne Human Research Ethics Committee.

One participant was excluded from analysis because of poor EEG signal quality. A second participant was excluded from analysis after a postexperiment debriefing revealed inadequate task understanding. For two other participants, computer error resulted in incomplete acquisition of EEG data. For these participants, behavioral analyses are reported only for task blocks in which complete EEG data were available (8 and 7 of 15 blocks, respectively). Final analyses were performed on data acquired from 16 participants (mean age, 22.63 years; age range, 18-29 years; 6 females).

In order to incentivize task performance, participants received monetary compensation for participation that was proportional to task winnings. Actual remuneration values were within the range of AUD $20-30 (mean remuneration, AUD $25.89; SD, AUD $4.36).

### Behavioral paradigm

**Figure 1.**
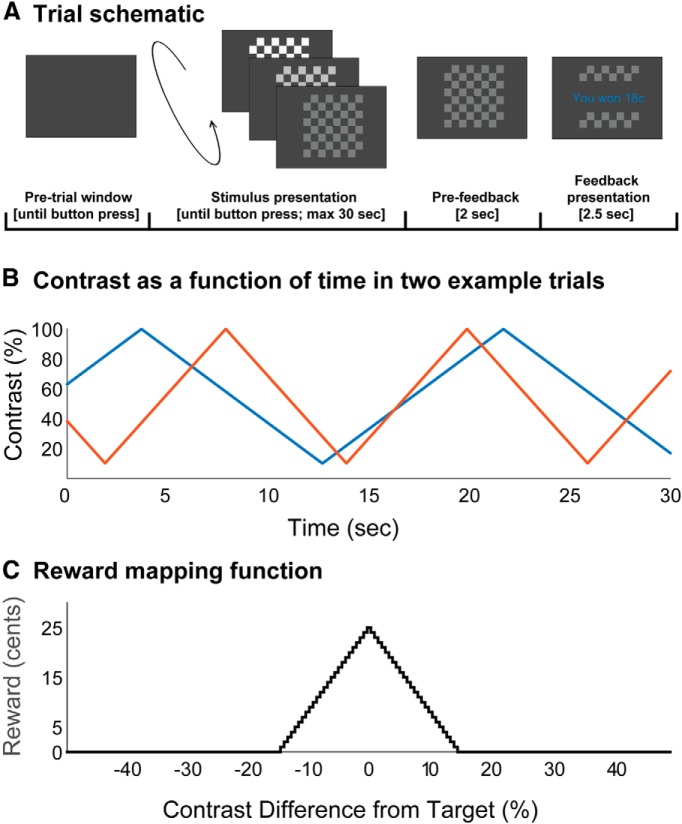
***A***, Following a self-paced button press, a checkerboard stimulus was presented whose contrast changed linearly. The participant could at any time select the contrast displayed on screen by pressing a button with the right index finger. The trial continued until a button was pressed or until stimulus duration exceeded 30 s. Following the participant’s choice, the selected contrast remained on screen for 2 s, after which time the monetary reward associated with the chosen contrast was displayed for 2.5 s. In the event that no button was pressed within 30 s, feedback was a message reminding the participant of the task instructions. ***B***, Two demonstrative examples of stimulus contrast as a function of elapsed time. Example trial 1 (blue) has an initial contrast of 63%, is initially increasing, and has a half-cycle period of 9 s. Example trial 2 (red) has an initial contrast of 39%, is initially decreasing, and has a half-cycle period of 6 s. The checkerboard stimulus phase reversed at a rate of 12 Hz. ***C***, Functional mapping between the contrast difference from target and monetary reward. The mapping was a symmetrical triangular function with a center of 0% contrast difference, a half-width of 15% contrast difference, and a height of 25 cents. As such, the received reward was maximal when the participant responded at the target contrast and decreased linearly with increasing difference of chosen contrast from the target. The reward was 0 for responses at >15% distance. Feedback received was rounded to the nearest whole-cent value.

Participants performed a novel perceptual learning task while EEG data were recorded. The task required participants to learn an arbitrary mapping between the contrast of a stimulus and monetary reward. This mapping was constant within each block, but differed between blocks. During each block, participants performed a number of consecutive trials in which they aimed to choose the contrast associated with the maximum reward (target contrast). The stimulus was a grayscale checkerboard stimulus (Fig. 1*A*), which was presented on each trial for a duration of up to 30 s. During this time, the contrast of the checkerboard changed linearly (Fig. 1*B*), and the participant could at any time choose the contrast displayed on screen by pressing a button with the right index finger. After choosing a contrast, participants received the reward associated with the chosen contrast. Crucially, the amount of reward that participants received for a given contrast was determined by the proximity of the chosen contrast to the maximally rewarding target contrast. Concretely, reward was assigned as a function of the difference between the chosen and target contrasts, and reward per trial was in the range 0–25 cents (rounded to the nearest integer value). The mapping (Fig. 1*C*) was a symmetrical triangular function with a center of 0% contrast difference, a half-width of 15% contrast difference, and a height of 25 cents. As such, the received reward was maximal when the participant responded at the target contrast, and decreased monotonically with increasing difference of chosen contrast from the target. The reward was 0 for responses at >15% distance. This relationship is formally expressed in Equation (1):

(1) $$R\left(r_{t},x_{t}\right)=\{\begin{array}{c} 25-\frac{(5||{\;r}_{t}-x_{t}||)}{3},\left|r_{t}-x_{t}\right|<15\\ 0,\left|r_{t}-x_{t}\right|\geq 15 \end{array}$$

where *t* is the trial number, *r_t_* is the target contrast on trial *t*, and *x_t_* is the participant’s chosen contrast on trial *t.*

By choosing different contrasts and obtaining associated rewards over a number of trials, participants were able learn the target contrast and thereby maximize their winnings. One important feature of the task was that participants were never informed of the exact contrast value they had chosen. As a result, there remained at all times a degree of uncertainty concerning contrast to which the observed feedback pertained.

The initial contrast and initial direction of contrast change were randomly determined on each trial using a Matlab random number generator with unique seeds for each participant. The half-cycle period, defined as the time required for the contrast of the checkerboard to change from one extreme to the other, was likewise randomly selected as 6, 7, 8, or 9 s on each trial in order to nullify the potential confound of learning based on temporal cues. The checkerboard phase reversed at a rate of 12 Hz, giving it a flickering appearance.

Prior to testing, participants received training to instruct them in the shape of the reward function and were informed that each block would have a different target in the range of 10-100%. Participants then completed 15 blocks of the task, each with a different target contrast, over approximately 60 min. Each block continued until the cumulative checkerboard presentation duration for the block exceeded 3 min, or until 25 trials were completed, whichever occurred sooner. As a result, the number of trials per block varied (mean, 18.46 trials; SD, 3.68 trials). This ensured that participants could not rush through the task, and that it was not possible to trade off experiment duration against monetary winnings. Finally, target contrasts were assigned subject to the constraint that the reward available for lowest and highest contrasts must be 0. In practice, because of the width of the reward distribution (Fig. 1), this meant that target contrasts were assigned on the interval [25, 85] rather than the interval [10, 100]. This ensured that the total reward available in each block was equivalent, and that feedback was always equally interpretable. Participants were not informed of this manipulation.

Stimuli were presented using a Sony Trinitron G420 CRT monitor at a framerate of 120 Hz. During task performance, participants were seated comfortably in a darkened room, using a chin rest at a distance of 77 cm from the screen. Checkerboard stimuli were 560 × 560 pixels in size, measuring 19.5 × 19.5 cm on the screen and subtending a visual angle of 14.43^°^ by 14.43^°^. Responses were recorded using a five-button Cedrus Response Box.

### EEG data acquisition

The electroencephalogram was recorded from 64 Ag/AgCl active scalp electrodes located according to the International 10-20 system. Electrodes interfaced with a BioSemi ActiveTwo system running ActiView acquisition software, and used an implicit reference during recording. Data were linearly detrended and re-referenced off-line to an average of mastoid electrodes. The vertical and horizontal EOGs were recorded from infraorbital electrodes that were horizontally adjacent to the left eye. The EEG was recorded at a sampling rate of 512 Hz. Using a linear finite impulse response filter, data were high-pass filtered at 0.1 Hz, low-pass filtered at 70 Hz, and notch filtered at 50 Hz to remove background electrical noise. Data were analyzed in epochs consisting of data from 1500 ms before to 1500 ms after the presentation of monetary feedback.

During preprocessing, data were first manually screened to exclude epochs contaminated by skin potential or muscle artifacts. Poor-quality data channels were then identified visually and corrected using the spline interpolation routine as implemented by the EEGLAB processing toolbox (Delorme and Makeig, 2004). An independent-components analysis, as implemented in the EEGLAB toolbox, was performed on the resulting dataset to identify and remove components related to eye movements and eye-blink artifacts. A final impartial artifact screening procedure was performed to exclude from analysis all epochs in which maximum/minimum amplitudes exceeded ±500 µV. Finally, a standard current source density (CSD) analysis was conducted on epoched EEG data for each of the 64 electrode sites using the CSD toolbox (version 1.1; Kayser and Tenke, 2006). This analysis calculates the spatial second derivative of voltage distribution over the scalp, and is a commonly applied procedure in the P3 and SPN literature (Gaeta et al., 2003; Catena et al., 2012). Spatial filters, such as CSD, are recommended for single-trial EEG analysis because of their ability to extract estimates of activity that are unique to each electrode, which increases the signal-to-noise ratio of individual trial CSD-ERPs, thereby augmenting the statistical power of analysis (Blankertz et al., 2008).

### Single-trial CSD-ERP calculation

Single-trial P3 amplitudes were calculated at the following four electrodes typically investigated in condition-based P3 ERP research: FCz, Cz, CPz, and Pz (Mecklinger and Ullsperger, 1993; Troche et al., 2009). These electrodes were chosen to allow investigation of the effects of belief update on the topographically distinct P3a (fronto-central) and P3b (parietal) subcomponents of the P3 (for review of P3 subcomponents, see Polich, 2007).

For each electrode, P3 amplitude was calculated as the maximum voltage in the window from 300 to 450 ms after feedback presentation. This window was chosen according to a consensus estimate of latency of the peak of the P3 (Polich, 2007) and accounted for trial-to-trial variability in P3 peak latency. Voltages at each electrode were baseline corrected to the mean voltage within the period from 0 to 200 ms prefeedback.

Single-trial SPN amplitudes were calculated at 10 electrodes typically investigated in condition-based SPN ERP studies: F3, F4, C3, C4, T7, T8, P3, P4, O1, and O2 (Kotani et al., 2003). This allowed the investigation of the relationship between belief uncertainty and SPN amplitude at bilateral frontal, central, temporal, parietal, and occipital electrodes. For each electrode, SPN amplitude was calculated as the mean voltage in the window from 0 to 500 ms prior to the presentation of feedback. This window was longer than that used in some previous studies (Kotani et al., 2003; Masaki et al., 2010; Catena et al., 2012), but this was considered necessary to stabilize the measurement volatility associated with the calculation of SPN amplitudes in single trials rather than from averaged waveforms. Voltages were baseline corrected at each electrode to the mean voltage within the period from 1300 to 1500 ms prefeedback.

### Overview of behavioral models

We estimated two competing behavioral models: an unbiased updating model and a win-stay lose-shift (WSLS) heuristic model. The updating model assumed that participants maintained a belief distribution over the entire range of possible contrasts and updated this distribution as feedback provided new information on each trial. By contrast, the WSLS model assumed that, rather than maintaining a full belief distribution across contrasts, choices exhibited a one-trial memory such that participants tried to repeat the choice of the previous trial if it had resulted in any reward, and shifted randomly to a new contrast otherwise. Both models are formally specified below.

Parameters were estimated for each participant with maximum likelihood estimation using the interior point algorithm as implemented in MATLAB (MathWorks). Standard statistical model comparison tools were used to identify which model provided the best account of observed choices. The best-fitting model from this comparison was used in subsequent analyses of ERP results.

### Unbiased updating model

For the unbiased updating model, a variant of a Bayesian grid estimator (Moravec, 1988) was used to obtain estimates of participants’ belief uncertainty and belief update size on each trial. In general terms, the model made a probabilistic estimate on each trial of participants’ beliefs regarding the level of the target contrast. These estimates could then be used to quantify (1) the degree of belief uncertainty in any given trial and (2) how beliefs changed from trial to trial as new feedback information was received.

Structurally, the model describes participants’ prior beliefs at each trial *t* by a probability mass function (PMF) θ*_t_* over a contrast space divided into *J* discrete bins 1, 2, 3, … *J*, such that the value of the PMF at each bin *j*, θ*_t_*(*j*), represented the subjective probability that the target contrast *r_t_* fell within bin *j* on trial *t*. Bins had a width of 0.61% contrast, which was chosen as the largest value sufficient to resolve different monetary feedback values. As a result, the belief distribution contained *J =* 148 contrast bins on the interval [10, 100]. At the beginning of each block, this distribution was initialized according to a discrete uniform distribution, reflecting participants’ a priori uncertainty regarding the target contrast. Use of an uninformative starting prior is consistent with the modeling protocols of similar studies (Mars et al., 2008; Ostwald et al., 2012). Except for transitions between one block and the next, beliefs were considered to be updated sequentially, such that the posterior distribution of trial *t* was the prior distribution for trial *t +* 1.

For each trial *t*, participants observed the feedback *f_t_*after the choice of contrast bin *x_t_*, which was determined according to the feedback mapping function *R* specified by Equation (1). Upon receipt of monetary feedback, the prior θ*_t_* was updated for each contrast bin *j* according to Bayes’ rule, as follows:

(2) $$\theta_{t+1}(j)=\frac{\theta_{t}(j)\mathrm{Pr}\text{(}f_{t},x_{t}\text{|}r_{t}\in j)}{\mathrm{Pr}(f_{t},x_{t})}$$

The left-hand side of Equation (2) is the value of the posterior belief distribution for bin *j*, calculated by multiplying the participant’s prior belief that the target contrast fell within bin *j*, θ*_t_*(*j*) by the likelihood of observing the choice/feedback pair if the target were in bin *j*, *Pr*(*f_t_*, *x_t_*|*r* ∈ *j*), and dividing by the marginal likelihood of the update *Pr(f_t_*, *x_t_*).

Importantly, in the task used in the present study, participants did not possess perfect knowledge of which contrast they had chosen (e.g., if the true value of a participant’s chosen contrast was 50%, the participant might know only that he or she had chosen some contrast between 40% and 60%). To account for this response uncertainty, the likelihood *Pr*(*f_t_*, *x_t_*|*r* ∈ *j*) in Equation (2) was expressed as a probability-weighted sum over all contrasts the participant might have believed he or she had chosen. As such, the likelihood was considered not at a single contrast value but over the set of all candidate contrast bins J*, J* = *J*, as follows:

(3) $$\mathrm{Pr}\text{(}f_{t},x_{t}\text{|}r_{t}\in j)=\sum_{J*}[\mathrm{Pr}\left(r_{t}\in j\text{|}f_{t},x_{j\text{*}}\right)\mathrm{Pr}\left(x_{t}=x_{j\text{*}}\right)]$$

For each candidate contrast j* in the set J*, the probability *Pr*(*r*∈*j*|*f_t_*, x_j*_) was equal to 1 if it was logically possible under the task feedback mapping for the target contrast *r* to belong to bin *j* if feedback *f_t_* was observed after a choice of contrast x_j*_, and was 0 otherwise. That is:

(4) $$\mathrm{Pr}\left(r\in j\text{|}f_{t},x_{j\text{*}}\right)=\{\begin{array}{c} 1,R(r_{\text{t}},x_{j\text{*}})=f_{t}\\ 0,R(r_{t},x_{j\text{*}})\neq f_{t} \end{array}$$

Each candidate contrast likelihood was then weighted by the subjective probability *Pr*(*x_t_* = x_j*_) that the chosen contrast *x_t_* was equal to the candidate contrast x_j*_. This subjective probability reflects participants’ response uncertainty and was calculated as the function *G_0_*, a 0 mean Gaussian function of the contrast difference between the true chosen contrast *x_t_*and the candidate contrast x_j*_, as follows:

(5) $$\mathrm{Pr}\left(x_{t}=x_{j\text{*}}\right)=G_{0}\left(x_{t},x_{j\text{*}},\sigma \right)\equiv \frac{1}{\sigma \sqrt{2\pi }}e^{-\frac{{\left(x_{t}-x_{j\text{*}}\right)}^{2}}{2\sigma^{2}}}$$

The SD σ of the distribution function reflects the degree of response uncertainty, such that greater values of σ result in more weight being given to candidate contrasts at a greater distance from the true chosen contrast. In the case of 0 response uncertainty, Equation (5) reduces to a Dirac δ function. Given Equations (3) and (5), Equation (2) can be rewritten:

(6) $$\theta_{t+1}(j)=\frac{\theta_{t}(j)\sum_{J\text{*}}\left[\mathrm{Pr}\left(r_{t}\in j\text{|}f_{t},x_{j\text{*}}\right)\frac{1}{\sigma \sqrt{2\pi }}e^{-\frac{{\left(x_{t}-x_{j\text{*}}\right)}^{2}}{2\sigma^{2}}}\right]}{\mathrm{Pr}(f_{t},x_{t})}$$

For an intuitive understanding of this model parameterization, consider the case of a participant who has perfect knowledge of exactly which contrast he or she has chosen. In this case, σ = 0 and *Pr*(*x_t_* = x_j*_) is equal to 1 where x_j*_ = *x_t_*, and 0 elsewhere. In this case, the likelihood in Equation (3) is calculated exclusively on the basis of the true chosen contrast, and the participant is able to make very precise inferences from the observed feedback. In the present study, it was considered highly unlikely that participants had perfect knowledge of their chosen contrast. By allowing σ to vary, the model allows that participants consider a range of alternative hypotheses concerning the chosen contrast when updating their beliefs. The parameter σ was permitted to vary between participants when fitting the unbiased updating model.

To implement this model, we made the further assumption that participants’ choices were determined by beliefs, such that contrast bins with a higher probability of containing the target contrast had a higher probability of being chosen, subject to the response uncertainty during choice. Formally, the PMF for contrast choices over the set of contrast bins *J* was determined by convolving the prior belief distribution θ*_t_* by the response uncertainty function *G_0_* over the set of contrast bins *J*, as follows:

(7) $$\mathrm{Pr}\left(x_{t}\right)=\frac{\left(\theta_{t}\text{*}G_{0})[J\right]}{k}$$

where *k* is a normalization constant ensuring that Σ*Pr*(*x_t_*) = 1.

As an illustration of how this model operates, we can assess the effects on belief of receiving feedback of *f_1_* = 20 cents after a choice of *x_1_* = 50% contrast on trial 1 (*t =* 1). For the sake of simplicity, rather than enumerating effects across the entire belief distribution, we consider the effects of observing this feedback on one contrast bin of the belief distribution centered around 60.2% contrast (*j =* 83). Since we are considering the first trial of a block, prior belief probability for this contrast θ_1_ (83) = 1J = 0.007. If we assume that the perceptual uncertainty parameter σ is equal to 15, then, by Equation (3), the likelihood *Pr*(20c, 50%|*r* ∈ 60.2%) is equal to 0.026. In order to calculate the posterior probability, we multiply the likelihood 0.026 by the prior belief probability 0.007 and divide by the marginal likelihood to normalize, giving θ*_2_*(83) = 0.013. By calculating the ratio of posterior and prior, we observe that the participant’s subjective belief that the target contrast falls within this bin has nearly doubled in strength as a result of the information provided by feedback: $\frac{\theta_{2}(j)}{\theta_{1}(j)}$ = $\frac{0.013}{0.007}$ = 1.86.

### Win-stay lose-switch heuristic model

Unlike the unbiased updating model, the WSLS model does not assume that participants maintain a belief distribution over the entire range of contrasts. Instead, this model predicted that participants’ behavior on a given trial was a function of whether or not they had received reinforcement on the preceding trial (Robbins, 1952). Specifically, the model assumed that participants attempted to repeat the contrast choice of the previous trial if they had received any monetary reward on the previous trial (win), subject to response uncertainty, or shifted randomly to a new contrast if they had not received monetary reward (loss) or at the start of a new block. This gives the following choice probability function:

(8) $$\mathrm{Pr}\left(x_{t}\in j\right)=\{\begin{array}{c} \frac{\left(\delta (j-x_{t-1})\text{*}G_{0})[J\right]}{k},f_{t-1}>0\\ \frac{1}{J},otherwise \end{array}$$

where *k* is a normalization constant. Equation (8) implements the win case with the convolution of the 0 mean Gaussian response uncertainty function given in Equation (5) with the Dirac delta function δ, which is equal to 1 at the contrast bin chosen in the previous trial contrast and 0 elsewhere. This allows for the WSLS model to account for response uncertainty in a fashion similar to that of the unbiased updating model, thereby ensuring that predicted choice probabilities are comparable across the two models.

### Calculation of belief updating variables

For the unbiased updating model, which assumed that participants updated a belief distribution across all contrasts, estimations of subjective belief distributions could be used to calculate the following three variables of interest on each trial: belief uncertainty prior to the receipt of feedback; postfeedback belief update size; and postfeedback surprise (Mars et al., 2008).

Belief uncertainty was calculated as Shannon entropy (Shannon, 1948) over contrast bins of the prior distribution, as follows:

(9) $$H\left(\theta_{t}\right)=-\underset{J}{\sum }\theta_{t}(j)\log_{2}\theta_{t}(j)$$

Shannon entropy was used as an uncertainty metric because the entropy *H* of a probability distribution represents the degree of uncertainty coded by that set of probabilities. The entropy of a distribution is equal to 0 only in the case of complete certainty, when all probabilities but one are 0. Conversely, the entropy of a distribution is maximal when all probabilities have an equal value, as in a uniform distribution. In the present study, therefore, higher entropy values of the belief distribution reflected greater levels of belief uncertainty.

Belief update size was calculated as the mutual information of prior and feedback. This quantity represents the degree to which uncertainty is resolved in the transformation from prior to posterior probabilities, and corresponds to the information content (*I*) of feedback: the more informative feedback is, the greater the reduction in uncertainty from prior to posterior beliefs. Accordingly, belief update size was calculated as the difference in entropy between prior and posterior beliefs, as follows:

(10) $$I\left(\theta_{t};x_{t},f_{t}\right)\equiv H\left(\theta_{t}\right)-H\text{(}\theta_{t}\text{|}x_{t},f_{t})=H\left(\theta_{t}\right)-H(\theta_{t+1})$$

This value was calculated for each trial and provided a model-based estimate of the degree to which feedback was used by participants to update their beliefs regarding the location of the target contrast in contrast space. Larger values of *I* indicate greater resolution of uncertainty, and therefore larger belief updates.

In addition, we note that in the literature, belief update size is sometimes also measured by a metric termed Bayesian surprise (Baldi and Itti, 2010; Ostwald et al., 2012), which can be calculated as the Kullback–Leibler divergence of prior and posterior. In order to allow comparison between the present study and previous research, Bayesian surprise, denoted *I_KL_* (Kullback–Leibler divergence), was also calculated as an alternative measure of belief update size, as follows:

(11) $$I_{KL}\left(\theta_{t},\theta_{t+1}\right)\equiv \underset{J}{\sum }\left[\theta_{t}\left(j\right)\ln\left(\frac{\theta_{t}\left(j\right)}{\theta_{t+1}\left(j\right)}\right)\right]$$

Finally, we calculated feedback surprise *S,* a measure of the improbability of observing a particular feedback value given a certain contrast choice under certain beliefs (Shannon, 1948). Formally, this was computed as the negative logarithm of the probability of observing a certain feedback value *f_t_* given the prefeedback belief distribution θ*_t_*, and the chosen contrast value *x_t_*:

(12) $$S\left(\theta_{t},f_{t},x_{t}\right)\equiv -\log_{2}\mathrm{Pr}(f_{t}\text{|}x_{t},\theta_{t}\text{)}$$

It has previously been shown that surprise was encoded in the amplitude of the P3 at parietal electrodes in a serial reaction time task (Mars et al., 2008), and this quantity was therefore calculated in order to allow us to dissociate any observed effects of belief updating from effects of surprise. Importantly, while there is a superficial conceptual resemblance between belief update size and surprise, the two quantities are mathematically distinct (Baldi and Itti, 2010). Feedback surprise relates to the probability of occurrence of a particular feedback value; it is calculated as a function of the prior predictive distribution over possible observations. By contrast, belief updating relates to the degree to which feedback causes beliefs to be modified, and is calculated as a function of the prior and posterior distributions over parameters. Moreover, it has been shown that the two quantities have distinct neural substrates, with belief updating encoded in anterior cingulate cortex (ACC) and surprise encoded in posterior parietal cortex (O’Reilly et al., 2013). Furthermore, from a statistical perspective, an important difference between surprise and belief updating is that belief updating is calculated as the distance measure between prior and posterior belief distributions, whereas surprise is calculated only at a single point in the prior distribution.

### Single-trial regression analysis of belief updating

Robust single-trial multiple regression analyses were used to investigate (1) the effect of feedback reward, feedback surprise, and belief update size on the amplitude of the post-feedback P3 component; and (2) the effect of belief uncertainty on the amplitude of the prefeedback SPN. To account for individual variability in the amplitude of ERP components, both P3 and SPN amplitudes were normalized on an individual-participant level prior to regression analysis. To account for heteroscedasticity in the relationship between model-derived belief variables and single-trial ERP amplitude estimates, robust (weighted least squares) linear regression analyses were used. For all ERP analyses, regressions were run separately for each participant at each electrode, and resulting β coefficients were subjected to Bonferroni-corrected single-sample *t* tests in order to determine whether the effect of each predictor significantly different from 0 across participants.

## Results

### Behavioral task

**Figure 2.**
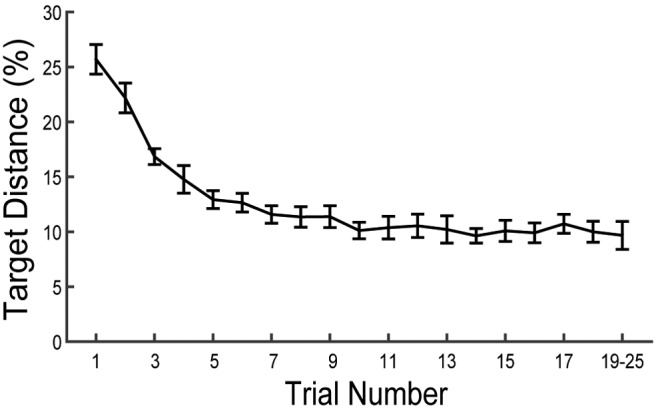
Mean accuracy as a function of within-block trial number across participants. Accuracy is presented as the absolute difference of chosen and target contrasts, where lower differences indicate better task performance. Error bars represent the SEM. Note that the number of trials per block varied across blocks and participants, and as a result some participants did not complete >19 trials in any block. This confound limited the interpretability of accuracy data for trial numbers >20, and the final data point of the series therefore represents mean accuracy across trials 19–25 for each participant.

Table 1 presents an overview of all statistical analyses reported. Across participants, responses became more precise with increasing within-block trial number (mean *β* = −0.65, *t*_(15)_ = −9.66, *p* = 0.00000008^a^), indicating acceptable task performance (Fig. 2). The mean absolute difference between the chosen contrast and the target contrast in the final trial of blocks was 9.24% (SD, 8.48%). This demonstrates that, while participants achieved proficiency on the task, their performance did not reach an absolute ceiling before block termination.

**Table 1.** Summary of statistical analyses

	Data structure	Type of test	Observed power
a	Normally distributed	Single-sample *t* test	1.0
b	Model likelihoods	BIC	Not applicable
c	Normally distributed	Single-sample *t* test	1.0
d	Normally distributed	Single-sample *t* test	0.54
e	Normally distributed	Single-sample *t* test	0.65
f	Normally distributed	Single-sample *t* test	0.06
g	Normally distributed	Single-sample *t* test	1.0
h	Normally distributed	Pearson correlation	0.99
i	Normally distributed	Single-sample *t* test	0.95
j	Normally distributed	Single-sample *t* test	1.0
k	Normally distributed	Repeated-measures ANOVA	0.77
l	Normally distributed	Repeated-measures ANOVA	0.08
m	Normally distributed	Repeated-measures ANOVA	0.13
n	Normally distributed	Single-sample *t* test	0.31
o	Normally distributed	Single-sample *t* test	0.97
p	Normally distributed	Single-sample *t* test	0.98
q	Normally distributed	Single-sample *t* test	1.0

### Model comparison

We used standard model comparison techniques in order to determine which of the two computational models described above provided the best account of participants’ choices. Table 2 presents Bayesian information criterion (BIC) values for the unbiased updating and WSLS models. Use of BIC allows us to identify models that account for data in a parsimonious way by balancing measures of parsimony (number of parameters) against measures of goodness-of-fit (log likelihood).

**Table 2.** Summary of behavioral model fits for 4417 choices by 16 participants

Model	Parameters per participant	Parameters	Belief distribution	Log-likelihood	BIC	*N* best fit
Unbiased updating	1	σ	Yes	−20190	40515	11
Win-stay/lose-shift	1	σ	No	−20350	40834	5

It can be seen that the unbiased updating model provided the best overall account of participants’ choices^b^. This model assumed that participants maintained a complete belief distribution over the contrast space and that belief updates were unbiased by the direction of contrast movement at the time of choice. Furthermore, examination of model fits for individual participants using participant-specific BIC values revealed that the unbiased updating model provided the best account of choices for a clear majority of participants (Table 2, *N* best fit column). As a result, all ERP analyses made use of belief variables calculated from the unbiased updating model.

### Computational model

**Figure 3.**
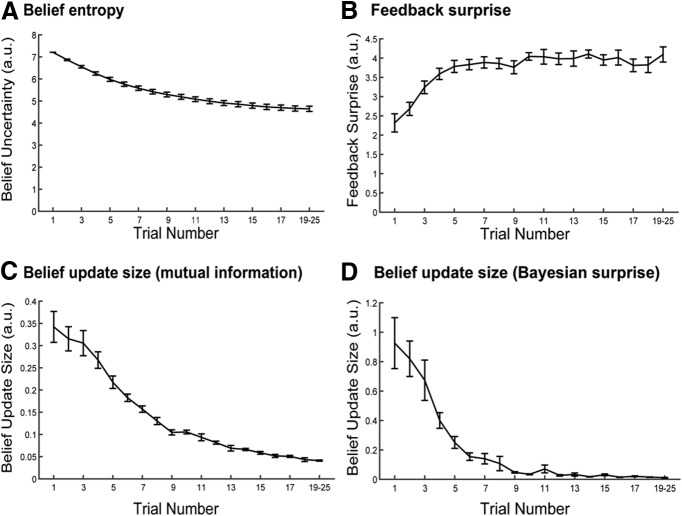
Computational belief variables as a function of trial number. ***A***, Belief entropy. ***B***, Feedback surprise. ***C***, Belief update size measured as mutual information (see Eq. 14). ***D***, Belief update size measured as Bayesian surprise (see Eq. 15). Note that the number of trials per block varied across blocks and participants, and, as a result, some participants did not complete >19 trials in any block. This confound limited the interpretability of computational belief variables for trial numbers >20, and the final data point of the each series therefore represents a mean across trials 19–25 for each participant. Error bars represent the SEM.

Across participants, pretrial belief uncertainty, as quantified by the unbiased updating model, was found to significantly predict choice accuracy on the upcoming trial (mean β = 5.71, *t*_(15)_ = 11.74, *p* = 0.000000006^c^. Moreover, model-estimated belief uncertainty predicted choice accuracy even after accounting for the effects of the following three linear and nonlinear trial number regressors: a linear term, a quadratic term, and a cubic term. In this analysis, we found significant effects for the quadratic trial number term (mean β = 0.12, *t*_(15)_ = 2.15, *p* = 0.048^d^) and the cubic trial number term (mean β = −0.004, *t*_(15)_ = −2.44, *p* = 0.03^e^), but not for the linear effect of trial number (mean β = −0.28, *t*_(15)_ = −0.42, *p* = 0.68^f^). However, even when accounting for these effects of trial number, the linear relationship between model-estimated belief uncertainty and choice accuracy was still strong (mean β = 9.75, *t*_(15)_ = 6.68, *p* = 0.0000007^g^*)*. This result indicates that belief uncertainty was predictive of choice accuracy even when linear and nonlinear trial-by-trial learning effects were accounted for, suggesting that the task model fit the data well and validating the use of variables derived from this model in single-trial regression analyses. Figure 3 presents descriptive statistics for each of the calculated belief variables as a function of trial number.

In the task model, participants’ response uncertainty was captured by the parameter σ, the SD of the Gaussian noise affecting the marginal likelihood of belief updates. Across participants, estimates of σ had a mean value of 12.99 (SD, 4.42), and fit values of σ were positively correlated with participants’ overall task performance, as measured by the average deviance between chosen and target contrasts (*r*_(16)_ = 0.86, *p* = 0.00002^h^). Individual differences in σ were therefore behaviorally relevant, such that individuals with less response uncertainty tended to respond closer to the target contrast on average. This further validates our use of the Bayesian grid estimator to represent participants’ beliefs.

### Single-trial regression analysis

#### P3

**Figure 4.**
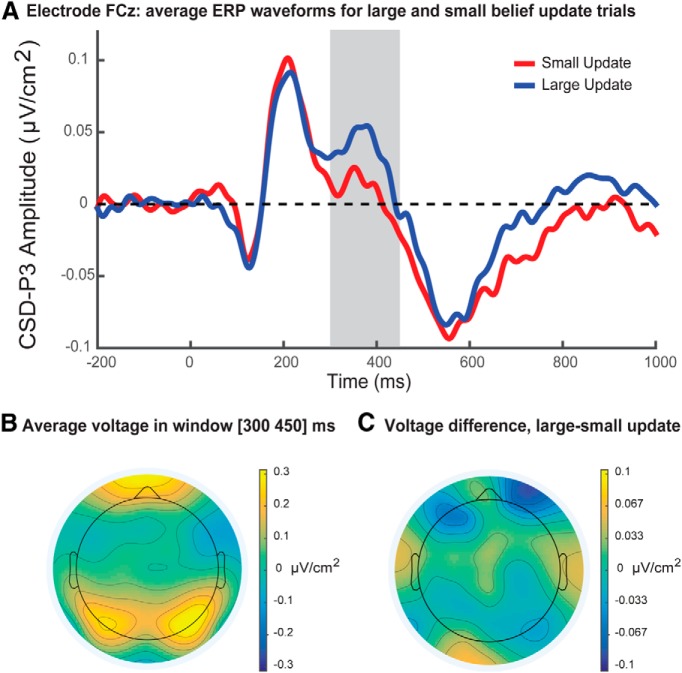
P3 analysis. ***A***, Median split waveforms for 200–1000 ms following visual presentation of feedback. The P3 regression analysis window is indicated by the gray bar. ERP waveforms were low-pass filtered at 30 Hz for display purposes only. ***B***, Mean voltage topography during the P3 analysis window from 300 to 450 ms following visual presentation of feedback (time = 0). ***C***, Topography of the mean voltage difference between large and small belief update trials across participants during P3 analysis window. A median split was used to divide trials into two bins for each participant, corresponding to large and small belief updates according to model-derived estimates. This median split was for display purposes only and was not used in the main regression analysis, which was based on single-trial amplitudes.

Single-trial regression analysis found a positive effect of belief update size (formally, the feedback-related reduction in entropy of the belief distribution approximated by a Bayesian grid estimator) on P3 amplitude at electrode FCz (mean β = 0.27, *t*_(15)_ = 3.33, *p* = 0.005^i^, Bonferroni corrected; Fig. 4). There was no effect of belief update size on P3 amplitude at electrodes Cz, CPz, or Pz, and no significant effect of reward magnitude or feedback surprise on P3 amplitude at any electrode. This indicates that single-trial amplitudes of the fronto-central P3a directly indexed model-derived measures of belief update size. Figure 4*B* displays the average voltage scalp distribution, and Figure 4*C* illustrates the difference map for large and small belief updates during the P3 time window. Table 3 displays a correlation matrix of the predictor variables included in the P3 regression analysis. Note that P3 regression analyses included either *I* or *I_KL_* as measures of belief update size, but never both.

**Table 3.** Correlation matrix for predictors in P3 regression analysis

	Reward	Belief update size (*I*)	Belief update size (*I_KL_*)
Reward	1		
Belief update size (*I*)	0.22 (0.19)	1	
Belief update size (*I_KL_*)	−0.24 (0.12)	0.64 (0.16)	1
Surprise	0.45 (0.21)	0.22 (0.12)	0.05 (.14)

Data are presented as mean Spearman coefficient across participants (SD).

As illustrated by Figure 3*C*, there was a significant tendency for belief update size *I* to reduce as the trial number increased (mean Spearman correlation across participants = −0.67, *t*_(15)_ = −17.91, *p* = 2 × 10^−11j^). As a result, we considered the possibility that the single-trial relationship between P3 amplitude and belief update size might have been confounded by an incidental effect of trial number on P3 amplitude. In order to address this possibility, we ran a control analysis in which trials were partitioned according to both trial number and belief update size. In this analysis, each trial was designated as an “early-,” “middle-,” or “late stage” trial, corresponding respectively to trial numbers 1–5, 6–10, and 11–15. Trials were also designated as either “small” or “large” belief updates according to a median split separately for each participant. We then used 3 × 2 repeated-measures ANOVA to assess separately the effects of trial number (early, middle, late) and belief update size (small, large) on mean P3 amplitudes at electrode FCz. Consistent with the single-trial regression results presented above, ANOVA results indicated a significant main effect of update size, *F*_(1,15)_ = 8.40, *p* = 0.01^k^, with large belief updates (mean = 0.053 μV/cm^2^, SD = 0.017 μV/cm^2^) associated with significantly larger P3 amplitudes than small belief updates (mean = 0.049 μV/cm^2^, SD = 0.016 μV/cm^2^). There was no main effect of trial number on P3 amplitude (*F*_(2,14)_ = 0.25, *p =* 0.78^l^), and no interaction between belief update size and trial number (*F*_(2,14)_ = 0.63, *p* = 0.55^m^). These results support the contention that fronto-central P3 amplitude indexed belief update size, and suggest that this effect was not confounded by any incidental effects of trial number.

Interestingly, there was no relationship between belief update size and P3 amplitude at any electrode when belief update size was calculated as Bayesian surprise *I_KL_* rather than mutual information *I* (mean β = 0.20, *t*_(15)_ = 1.54, *p* = 0.14^n^). This appears to suggest that the observed effects are specific to the mutual information formulation of belief update size. Note that regression analyses were each run with either *I* or *I_KL_* as measures of belief update size, never both.

Across participants, the mean P3 peak latency at electrode FCz was 338.43 ms (SD = 5.29 ms). There were no effects of reward, belief update size, or surprise on P3 peak latency at any electrode assessed.

#### SPN

**Figure 5.**
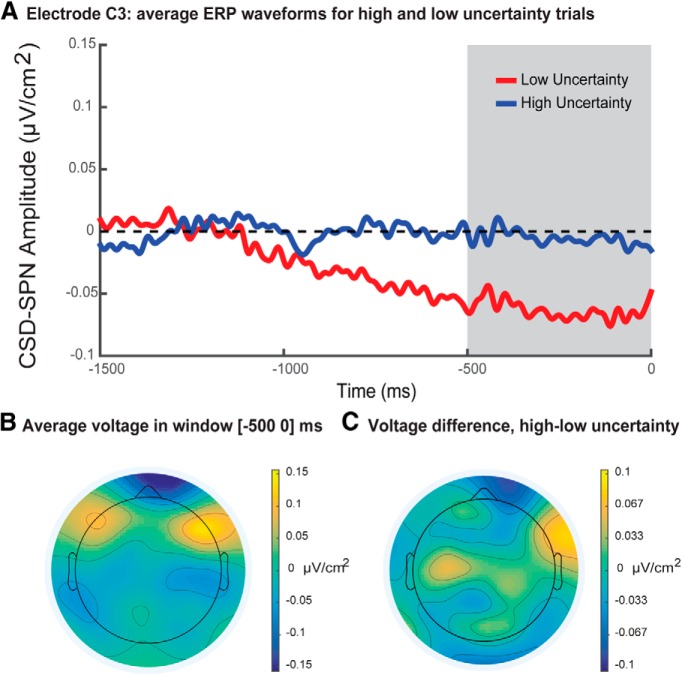
Stimulus-preceding negativity analysis. ***A***, Median split waveforms for 0–1500 ms prior to the visual presentation of feedback. The SPN regression analysis window from 0 to 500 ms preceding feedback is indicated by the gray bar. ERP waveforms were low-pass filtered at 30 Hz for display purposes only. ***B***, Mean voltage topography during SPN analysis window from 0 to 500 ms prior to visual presentation of feedback (time = 0). ***C***, Topography of the mean voltage difference between high and low uncertainty trials across participants during the SPN analysis window. A median split was used to divide trials into two bins for each participant, corresponding to high and low belief uncertainty according to model-derived estimates. This median split was for display purposes only and was not used in the main regression analysis, which was based on single-trial amplitudes.

Single-trial regression analysis found a small but significant negative effect of belief uncertainty (formally, the entropy of the belief distribution approximated by a Bayesian grid estimator) on SPN amplitude at electrodes C3 (mean β = −0.06, *t*_(15)_ = 3.56, *p* = 0.003^°^, Bonferroni corrected; illustrated in Fig. 5) and P4 (mean β = −0.05, *t*_(15)_ = 3.77, *p* = 0.002^p^, Bonferroni corrected). Note that SPN regression analyses were run including belief uncertainty as the sole predictor variable.

This result indicates that higher levels of belief uncertainty were associated with smaller SPN components. That is, the more certain participants were regarding the location of the target contrast in contrast space, the greater the amplitude of the SPN evoked in anticipation of feedback stimuli. Figure 5*B* displays the average voltage scalp distribution, and Figure 5*C* illustrates the difference map for high and low uncertainty during the SPN time window. There was no significant effect of belief uncertainty on single-trial SPN amplitude at any other electrode. However, as with belief update size, there was a strong negative correlation between belief uncertainty and trial number (mean Spearman correlation = −0.94, *t*_(15)_ = −115.20, *p* = 2 × 10^−23q^; Fig. 3*A*), as would be expected in a task in which participants learned incrementally from each trial. The strength of this relationship precluded a factorial control analysis to dissociate the effects of belief uncertainty and trial number on SPN amplitudes.

## Discussion

This study combined single-trial analysis of ERPs with computational modeling of belief. Our results showed that two mathematically distinct belief variables—update size and uncertainty—were encoded in distinct ERP components in a perceptual learning task. The combination of methods that we used linked the fine-grained information contained in single-trial EEG data with model-based estimates of participants’ latent beliefs, which would have been inaccessible to explicit testing. Our results suggest that trial-by-trial variations in the P3 and SPN reflect fundamental and distinct neural processes by which beliefs regarding the structure of the environment change over time.

Participants performed a simple perceptual learning task in which they learned a functional mapping between stimulus contrast and monetary reward. The task was both naturalistic and challenging: even with extensive practice, participants’ performances did not reach a ceiling, suggesting that participants continued to update beliefs throughout the experiment. We used a probabilistic model, termed the unbiased updating model, to infer participants’ beliefs at each trial from their choice history and found that model-based estimates of belief uncertainty predicted future choices well. The unbiased updating model gave better predictions of behavior than a competing model assuming a win-stay/lose-switch choice process in which participants chose on the basis of reward received on the previous trial rather than updating a full belief distribution.

We used the unbiased updating model to quantify the following three latent belief variables: belief uncertainty, belief update size, and surprise (Mars et al., 2008; Baldi and Itti, 2010; O’Reilly et al., 2013). We then investigated how the estimates of belief of the model update size and belief uncertainty were encoded in the P3 and SPN components of the ERP, respectively.

At the fronto-central midline electrode FCz, we found a significant positive relationship between postfeedback belief update size and single-trial P3 amplitude. This indicates that larger P3 amplitudes were observed in trials where feedback caused larger belief updates. Variability in single-trial P3 amplitude was best explained by regression using a model-derived estimate of belief update size and could not be accounted for by alternative regressors such as reward amount or feedback surprise. This is consistent with the hypothesis that P3 amplitude reflects a Bayesian belief-updating mechanism (Kopp, 2008; Mars et al., 2008). This theory attributes variability in P3 amplitude to the engagement of cognitive processes for revising internal models of the environment and predicts that larger updates to beliefs will be associated with larger P3 amplitude. Our study, using a single-trial regression approach, allowed for a direct test of this hypothesis, and our results provide broad support for the theory. In addition, we note that the observed association between belief update size and P3 amplitude disappeared when Bayesian surprise, rather than mutual information, was used as a measure of belief update size. The reason for this discrepancy is unclear, but may be related to differences in statistical power associated with the different temporal dynamics of the two measures (Table 1, Fig. 3). Other metrics, including a free-energy theoretical quantity termed model adjustment, have also been used in the ERP literature (Lieder et al., 2013). Future research should seek to provide a unifying account of belief updating by investigating circumstances under which these different metrics make differing cognitive and behavioral predictions.

The significant single-trial relationship between belief update size and P3 amplitude was restricted to a fronto-central midline electrode, with no evidence for a comparable effect at centro-parietal midline electrodes. This partition corresponds to a distinction drawn between the following two subcomponents of the P3: the fronto-central P3a and the centro-parietal P3b (Polich, 2007). In the present study, the P3a, but not the P3b, was an index of belief update size. In this regard, it is of particular interest that a previous study by Mars et al. (2008) found that feedback surprise, but not belief update size, was encoded in the P3b subcomponent, leading the authors to speculate that the P3a component may encode update size but not surprise. This proposal received empirical support from our findings. The observed results are broadly consistent with recent research investigating Bayesian single-trial properties of the P3 in a prediction task without reinforcement (Kolossa et al., 2015). Furthermore, the dissociation between frontal encoding of belief update size and parietal encoding of surprise is consistent with evidence from functional magnetic resonance imaging research. O’Reilly et al. (2013) measured brain activity during a saccadic eye movement task, and found that, whereas belief update size was encoded in ACC, surprise was encoded in posterior parietal cortex. Convergent methodologies, therefore, have shown that belief update size is encoded in both ACC and in the fronto-central P3a component of the ERP. Since the ACC has been proposed as a possible source of the P3a (Volpe et al., 2007), these results may be manifestations of the same underlying process. However, we note that since we did not use a standard P3a paradigm with novel nontarget distractors, it is possible that the P3a component encoding belief update size in the present study might also simply be labeled an anterior P3. To date, this nomenclature remains ambiguous (Luck, 2005; Polich, 2007).

A link between the P3 and belief updating has the potential to unify a number of disparate experimental findings. Larger P3 potentials are elicited by infrequent stimuli (Sutton et al., 1965), novel stimuli (Friedman et al., 2001), and stimuli imparting information (Sutton et al., 1967). Since these manipulations each vary the extent to which participants must revise an internal model of the environment, belief updating might be considered a general principle linking each of these observations. Moreover, the Bayesian perspective is broadly compatible with context-updating theory, which proposes that P3 amplitude reflects the revision of schemata concerning stimulus context (Donchin and Coles, 1988). Prior beliefs in the Bayesian sense are conceptual cognates of context schemata, and belief updating equivalent to schema revision. Of course, a Bayesian framework cannot account for all manipulations that affect P3 amplitude (Kopp, 2008). Other important manipulations include effects of stimulus value (Begleiter et al., 1983; Sato et al., 2005) emotional salience (Johnston et al., 1986), and target/nontarget status (for review, see Squires et al., 1975). The triarchic model of Johnson (1986) suggests that both the transmission of information (analogous to the effect of a Bayesian belief update) and stimulus meaning contribute to the amplitude of the P3. Since stimulus meaning was not manipulated in the present study, we are unable to assess how its effects might have interacted with observed effects of belief updating. Integrating these manipulations is a task for future research.

The present study also observed a significant negative relationship between belief uncertainty and prefeedback SPN amplitude. At electrodes C3 and P4, larger SPN components were observed in trials in which participants’ beliefs were more certain. The SPN has previously been linked to the anticipation of feedback that provides response reinforcement (Damen and Brunia, 1994). The left central electrode C3 was situated over primary motor cortical areas responsible for the right index finger button press that indicated participants’ choices. The observed association between uncertainty and SPN amplitude at C3 may therefore reflect motor learning, since preparatory neural activity in motor cortex is known to be associated with rapid visuomotor learning (Muellbacher et al., 2001; Paz et al., 2003). Likewise, encoding of belief uncertainty at electrode P4 may reflect anticipatory prefeedback processing, which is consistent with previous studies showing involvement of parietal SPN in reward processing (Kotani et al., 2003). However, we note that, whereas the present study found a negative association between SPN amplitude and uncertainty, one recent study (Catena et al., 2012) found a positive effect at frontal electrodes. Of course, it is problematic to compare frontal with central and parietal SPN, since different regions are likely to be recruited in different cognitive processes. Nevertheless, an important difference between the present study and that of Catena et al. (2012) pertains to the operationalization of uncertainty. We used a task in which uncertainty was reducible: with practice, participants could become more certain about the contrast–reward mapping. By contrast, Catena et al. (2012) tested irreducible uncertainty by varying cue–outcome association strength. The resultant use of different cognitive processes may explain the discrepancy between electrophysiological findings. Furthermore, we note that the SPN is generally elicited only during the period prior to the occurrence of a stimulus. As such, our finding that SPN amplitude indexes uncertainty is specific to the case of temporal anticipation and does not necessarily fully define a general principle of the neural encoding of uncertainty. Future research should seek to determine how belief uncertainty is encoded when there is not a well defined future time at which uncertainty will be resolved.

In the P3 analysis, an additional factorial control analysis demonstrated that single-trial regression results were unlikely to have been affected by the possible confound of trial number. In the SPN analysis, by contrast, since a relationship between trial number and belief uncertainty was an inherent feature of the learning task used in the present study, it was not possible to rule out a possible mediating effect of trial number on the relationship between SPN amplitude and belief uncertainty. Further research is required to determine whether the relationship between SPN amplitude and belief uncertainty holds even when uncertainty is not monotonically decreasing as a function of trial number.

In the present study, our intention was not to give a complete overview of the ERP correlates of feedback processing, but rather to investigate the role in belief updating of two particular ERP components (the P3 and SPN) that have been implicated in belief updating by past research. Indeed, the general neural response to feedback is likely to recruit many processes other than just those associated with the P3 and SPN, and research using different experimental tasks from the present study has identified other ERP components involved in learning from feedback. In particular, a large body of research suggests the importance of the feedback-related negativity (FRN; Miltner et al., 1997). This component has been strongly linked to the evaluation of feedback outcomes (Yeung and Sanfey, 2004; Achtziger et al., 2015) and has been theorized to index the magnitude of a reward prediction error associated with reinforcement learning (Holroyd and Coles, 2002). Given this theory, in the present study we would have expected the FRN to encode not the size of a belief update, or the uncertainty of beliefs per se, but the valence of feedback outcomes relative to participants’ expectations. This is conceptually a separate aspect of learning from the model-based definition of belief updating used in the present study. Furthermore, a recent review (Luft, 2014) noted that it is problematic to investigate the FRN in tasks such as that used in the present study, in which reward and performance feedback are delivered concurrently. Since the task used in the present study was not optimized for the investigation of the FRN component, we chose to exclude the FRN from our model-based single-trial regression analysis. Future research should investigate the interaction of the FRN with the ERP components identified in the present study by making use of a belief-updating task in which reward and performance feedback are orthogonal.

Finally, we note that while we assessed belief updating within a Bayesian framework, there is evidence that humans also perform non-Bayesian belief updating in some circumstances (Hogarth and Einhorn, 1992; Stern et al., 2010). We do not make the strong claim that all neural computations underlying perceptual learning take place according to Bayesian principles; instead, it is likely that the ability of decision makers to make use of Bayesian updating is constrained by the complexity of the decision situation and by the availability of heuristic alternatives to Bayesian updating (Achtziger et al., 2014, 2015). However, the results of the present study show that a Bayesian updating model outperformed a non-Bayesian heuristic model for a relatively simple perceptual learning task. Non-Bayesian belief updating may have distinct ERP correlates in more complex environments, as suggested by Achtziger et al. (2014, 2015), and further research is required to reconcile these perspectives.

In summary, the present study provides evidence that single-trial EEG data can be used to track the evolution of latent states of belief in humans. Our results build an empirical bridge between general theories of belief updating in cognition and a long tradition of research into the functional significance of ERPs. More broadly, our findings are a novel demonstration of the value and viability of computational cognitive modeling in EEG research.

## Synthesis

The decision was a result of the Reviewing Editor Lila Davachi and the peer reviewers coming together and discussing their recommendations until a consensus was reached. A fact-based synthesis statement explaining their decision and outlining what is needed to prepare a revision is listed below. The following reviewer(s) agreed to reveal their identity: Laurence Hunt

As you can see from the reviewer's comments below, both reviewers felt the manuscript was novel and important as little is known about the neural correlates of belief updating. The manuscript is well written, data analysis approach is clear and the take-home message is informative. Both reviewers suggest important revisions however (see below). Reviewer 1 would like you to consider whether computational model variables still account for choice accuracy when non-linear effects for trial numbers are examined. I also believe this would make a nice addition to the current analyses. Reviewer 1 also has some other minor suggestions. Reviewer 2 mainly would like you to expand the context of your findings to include other related work in the field. Please respond to all of the reviewer's comments in your revision.

Reviewer 1:

This manuscript seeks to capture features of the EEG that reflect different aspects of statistical learning - the uncertainty over beliefs, and the degree to which beliefs are updated. Although several possible models could have been used, the manuscript does so with use of a Bayesian learning model, as has become popular in this field in recent years. The manuscript is clearly written and the data appear to have been well analyzed, with clear take-home conclusions. I had a few general comments that I thought might improve it further.

1. The authors dismiss the idea that the relationship between model measures and choice accuracy can be explained away trial number, by including trial number as a coregressor of no interest. However, given the relationship between accuracy and trial number is non-linear (figure 2), this is rather unsurprising - a linear effect of trial number is unlikely to accurately capture choice accuracy. However, I suspect that including higher order terms of trial number (e.g. a quadratic term, i.e. trial number squared, and perhaps a cubic) *would* allow for a decent fit to the data shown in figure 2. The interesting question then is - do the computational model variables still account for choice accuracy when the non-linear effect of trial number has been accounted for?

2. I think this reflects a more general concern that many readers will have about the EEG effects shown. All of the computational model effects that are shown have a dependency upon trial number. Given that this is the case, can the authors exclude linear/non-linear effects of trial number as possible confounds in the EEG response?

3. The non-significant relationship of P3 with the KL divergence, given that the mutual information (MI) is highly correlated with it and *does* have a significant relationship, is a little puzzling (as the authors acknowledge). It would help if the non-significant effect size (beta value and T-statistic) was reported for the KL divergence nonetheless. It might also be interesting to test whether the portion of MI that is *orthogonal* to the KL divergence is the part that explains variability in P3 amplitude.

4. I was unconvinced that it was necessary to include the biased updating model, given that it failed to describe subject behaviour - it sounded like a nice idea that didn't work in practice, but I felt that it slightly muddied the waters to include it in the paper.

5. In the introduction, I didn't really agree with the discussion of the modelling as 'implementational' rather than 'algorithmic' - my reading of Marr would be that the kind of Bayesian model used by the authors is exactly what is meant by an 'algorithmic' model, and it is this model that is being used to describe the data; by contrast, an 'implementational' model would be one that describes the mechanisms by which Bayes rule is implemented at the level of cells, circuits and synapses - something that the current paper doesn't really speak to.

Reviewer 2:

Review on the manuscript Single-trial even-related potential correlates of belief updating submitted to eNeuro.

This manuscript presents a study on an interesting topic that has only rarely been investigated, yet. The present research is innovative and contributes very well to neuroscientific research on Bayesian updating processes. Three behavioral models were tested. An unbiased updating model, a biased updating model, and a win-stay lose-shift heuristic model. Participants completed a perceptual learning task while electrocortical activity was recorded. It was found that the P3 and the SPN amplitudes were related to belief update size (P3) and belief uncertainty (SPN).

All in all, I think that the paper makes a good contribution to the literature on neural processes underlying Bayesian updating. However, I have some concerns that should be addressed in a revision.

The paper is well written and easy to follow. The introduction refers to literature on Bayes updating processes in human cognition and emphasizes the importance of these processes in human decision making. Critical literature on whether human decision makers are able to use Bayes' rule (instead of just following simple heuristics), however, is only very shortly mentioned in the discussion, and not at all in the introduction. In order to get the whole picture under which conditions people will make rational decisions (in the sense of following Bayes' rule), this literature should also be integrated in the introduction (e.g., Achtziger Alós-Ferrer, Hügelschäfer, and Steinhauser, 2014 and 2015, SCAN, should be referred to). Otherwise, the paper's message would imply that people are generally good in belief updating, while in reality, this strongly depends on the decision situation (especially its complexity) itself. In situations in which they are confronted with situations in which a simple heuristic( representativeness, or reinforcement; see Achtziger et al., 2014, 2015, SCAN) conflicts with Bayes' rule most people use the simple heuristic and do not follow the Bayes' rule (this requires more effort, takes longer etc). Hence it is no surprise that in more simple situations in which there is no conflict between a simple and a complex rule to follow, Bayes Updating can be observed. This should at least be mentioned in the revised manuscript.

The author(s) concentrated on the P3 (and its components) and the SPN. Of course, there are many arguments that support the view that the P3 might be worth exploring in the context of belief updating (the same is true for SPN). However, there is a lot of research on the FRN (e.g., Holroyd &amp; Coles, Yeung &amp; Sanfey, Miltner; Achtziger, Alós-Ferrer, Hügelschäfer, and Steinhauser, 2015, SCAN ) that shows that deviations from expectations are strongly indicated by the ERP. This literature is completely left out of the manuscript. Referring to this literature on the FRN in decision making would improve the paper a lot, as it is closely linked to the research aim of the present experiment. Readers would get the whole picture on neural processes of decision making that requires Bayesian updating more easily if they were informed about this previous research.Similary, the author(s) should explain whey they did not analyze the FRN since participants were presented with clear feedback after their decision. This must have generated a strong FRN (especially after negative feedback) and should have provided more insights into learning processes in the present study. In a revised version of the manuscript, it should be explained why the FRN as an indicator of reinforcement learning was not analyzed.
